# Uncoupling of nutrient sensing and cell size control by specific defects in ceramide structure

**DOI:** 10.1242/bio.062633

**Published:** 2026-05-26

**Authors:** José Ignacio Quesada-Márquez, Ana Serrano, María Alcaide-Gavilán, Rafael Lucena

**Affiliations:** Department of Cell Biology, University of Seville, Seville 41012, Spain

**Keywords:** Ceramides, Cell growth, Cell size, Yeast, TORC2

## Abstract

Ceramides are essential structural lipids whose chemical diversity arises from variations in acyl-chain length and sphingoid-base modifications, yet how these structural features couple metabolic state to growth regulation remains unclear. In *Saccharomyces cerevisiae*, the target of rapamycin complex 2 (TORC2)–Ypk1/2 signaling axis coordinates plasma membrane homeostasis with cellular growth; however, the lipid-derived signals modulating this pathway are not fully defined. Here, we establish that the elongation of very long-chain fatty acids (VLCFAs), specifically to C_26_, is a critical determinant of the nutrient-dependent regulation of TORC2 activity. Based on a molecular caliper model for acyl-chain determination, we show that the TORC2–Ypk1 axis is specifically tuned to detect the successful completion of C_26_-VLCFA synthesis. Disrupting VLCFA elongation (*elo3*Δ) triggers constitutive TORC2 hyperactivation and a failure to reduce cell size in response to nutrient limitation. By expressing mammalian ceramide synthases (CerS1–CerS4), we demonstrate that TORC2 nutrient sensing is specifically tuned to acyl-chain length. While CerS1, CerS3, and CerS4 restore the rapid, nutrient-induced downregulation of TORC2, CerS2 expression phenocopies the *elo3*Δ mutant, exhibiting a total kinetic failure to inhibit TORC2 signaling upon nutrient shift. Notably, cells producing C_18_ ceramides (GhLag1) maintained size control despite elevated TORC2 activity, revealing that ceramide-dependent signaling intensity and the physical execution of size regulation can be uncoupled. We further demonstrate that while sphingoid-base hydroxylation is required for the execution of size remodeling, it is dispensable for nutrient sensing; *sur2*Δ mutants exhibited severe size defects despite maintaining statistically normal, nutrient-responsive TORC2 signaling. Overall, our findings reveal a functional hierarchy where the protein-mediated caliper measurement of VLCFA length serves as the primary sensor for TORC2 nutrient responsiveness, while subsequent lipid modifications govern the biophysical execution of cell size control.

## Teaser

**Summary:** We identify a C26-VLCFA ‘molecular caliper’ required for TORC2 nutrient-sensing, showing that ceramide acyl-chain length and hydroxylation independently regulate growth signaling intensity and the biophysical execution of cell size.

## INTRODUCTION

Cellular growth and size homeostasis depend on the coordinated expansion of the plasma membrane and the synthesis of its constituent lipids. In budding yeast *Saccharomyces cerevisiae*, this coordination is governed by the target of rapamycin complex 2 (TORC2), a conserved protein kinase complex that serves as a master regulator of plasma membrane tension and lipid biosynthesis (Aronova et al., 2008; Emmerstorfer-Augustin and Thorner, 2023; Lucena et al., 2018; Powers et al., 2010; Riggi et al., 2020; Roelants et al., 2017; Thorner, 2022). TORC2 signaling is tightly coupled to sphingolipid metabolism; when sphingolipid levels are low or the membrane is stressed, TORC2 is activated to phosphorylate its downstream effectors, the AGC kinases Ypk1 and Ypk2 (Berchtold et al., 2012; Lucena et al., 2018; Roelants et al., 2011). These kinases, in turn, stimulate ceramide synthase activity and upregulate sphingolipid production, restoring membrane homeostasis (Lucena et al., 2018; Muir et al., 2014; Roelants et al., 2011).

Sphingolipid metabolism and ceramide synthesis are poorly understood. Sphingolipids, sterols, and glycerophospholipids constitute the primary lipid components found in eukaryotic membranes, with a particular abundance in the plasma membrane. Ceramide is not only a building block of sphingolipids but also a critical signaling molecule, in all eukaryotic organisms, which regulates numerous cellular processes such as the cell cycle, cell growth and cell size maintenance (Alonso and Goñi, 2018; Chalfant and Del Poeta, 2010; Flor-Parra et al., 2022; Hannun and Obeid, 2018; Summers et al., 2019). For example, ceramides can cluster into ceramide-rich ordered domains at different membranes, serving as dynamic signaling hubs of the cell (Alonso and Goñi, 2018; Rodriguez-Gallardo et al., 2020). Misregulation of ceramide synthesis is associated with various diseases, including cancer (Alizadeh et al., 2023; Morad and Cabot, 2013), cardiovascular disease (Chaurasia and Summers, 2021; Green et al., 2021) or infections of opportunistic pathogens (Kumar et al., 2021; Urbanek et al., 2022).

Sphingolipid biosynthesis begins in the endoplasmic reticulum (ER) with the condensation by a serine palmitoyltransferase (SPT) of serine and palmitoyl-CoA to form 3-ketodihydrosphingosine, which is rapidly reduced to the long-chain base (LCB) dihydrosphingosine (DHS) (Beeler et al., 1998). Subsequent to the LCB formation, the pathway divides into two distinct arms, the dihydro (non-hydroxylated) and the phyto (hydroxylated) branches. The C4-hydroxylase Sur2 is the enzyme controlling the levels of these two branches (Haak et al., 1997). Ceramide synthase subunits Lac1 and Lag1 catalyze N-acylation of DHS to a very long-chain fatty acid (C_26_-VLCFA), producing dihydroceramide (DHCerA) (Guillas et al., 2001; Schorling et al., 2001; Vallée and Riezman, 2005). DHCer is hydroxylated at C4 by Sur2 to give phytoceramide (PHCerB). Alternatively, Sur2 can hydroxylate DHS to form PHS, which is then amide-linked to the same C_26_-VLCFA to yield PHCerB (Grilley et al., 1998; Haak et al., 1997). Thus, PHCer can be generated through two distinct routes: either via the hydroxylation of DHS to PHS followed by N-acylation or through the direct C4-hydroxylation of DHCer by Sur2. DHCer and PHCer can also be hydroxylated at the C2 position of the acyl chain by Scs7 (Dunn et al., 1998; Haak et al., 1997) to generate DHCerB or PHCerC. Finally, the polar head of ceramides can be further modified at the Golgi to generate complex sphingolipids (Funato and Riezman, 2001). These include inositol phosphoceramide (IPC), mannosylinositol phosphorylceramide (MIPC) or mannosyldiinositol phosphorylceramide (MIP_2_C).

**Fig. 1.**
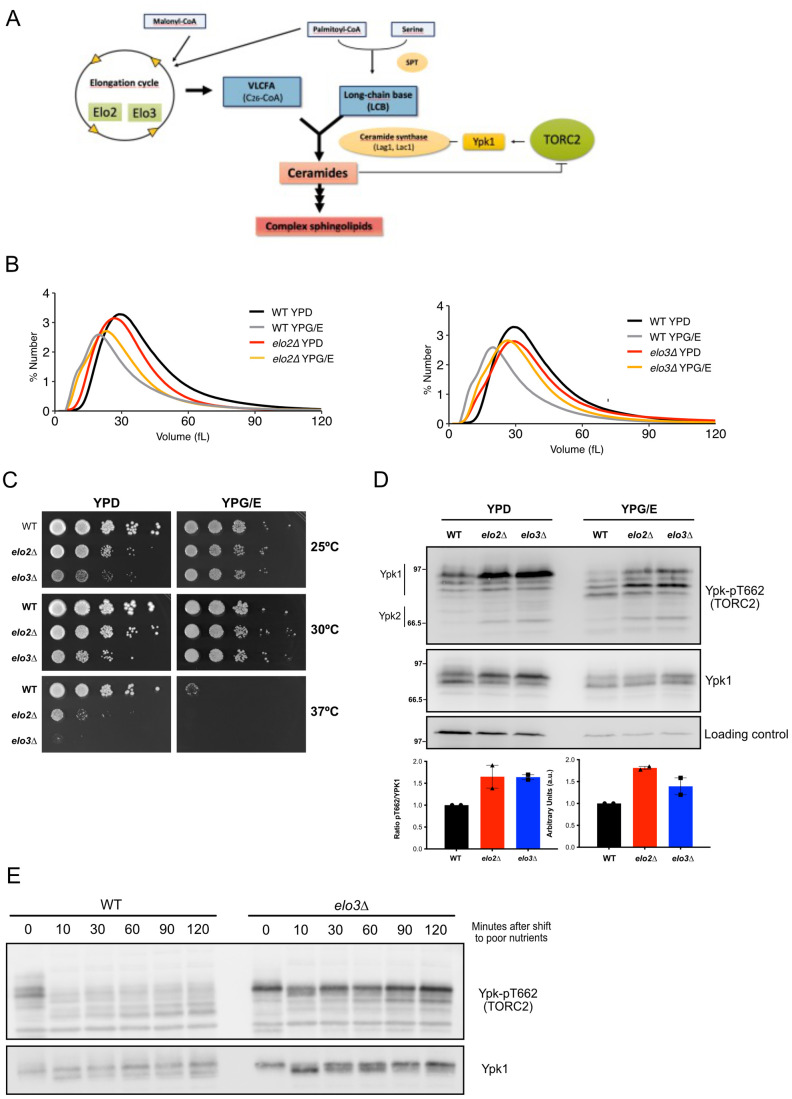
**Elo2- and Elo3-dependent VLCFA elongation modulates nutrient-responsive cell size and TORC2 signaling.** (A) Schematic representation of the fatty acid elongation pathway in *S. cerevisiae*. Elo1 elongates C_12_–C_16_ fatty acids, Elo2 extends C_16_–C_18_ species to C_24_, and Elo3 catalyzes the final step to generate C_26_-VLCFAs utilized by ceramide synthases Lag1 and Lac1. (B) Cells of the indicated genotypes were grown to log phase at 25°C, and cell size distributions were determined using a Coulter counter. *elo2*Δ cells exhibit a modest size reduction, whereas *elo3*Δ cells fail to downshift their size in poor carbon conditions. Each plot is the average of three biological replicates. (C) A series of tenfold dilutions of the indicated strains were grown on YPD or YPG/E plates at 25°C, 30°C and 37°C. (D) Cells of the indicated genotypes were grown at 25°C to log phase in YPD or YPG/E medium. Ypk-pT662 and total Ypk1 were assayed by western blotting. Asterisks indicate nonspecific background bands used as loading controls. Relative levels of Ypk1 phosphorylation at T662 were analyzed in WT, *elo2*Δ, and *elo3*Δ strains. To assess the capacity for nutrient-dependent downregulation, signals in poor media (YPGE) were normalized to the WT level in the same condition. Both *elo2*Δ and *elo3*Δ mutants exhibit persistent TORC2 signaling despite nutrient limitation, with *elo2*Δ showing a statistically significant failure to downregulate pT662 levels. Data represent the mean±s.e.m. of two independent biological replicates. Statistical significance was determined by one-way ANOVA (see main text for details). (E) WT and *elo3*Δ cells were grown to exponential phase in YPD and shifted to YPGE. Protein samples were collected at the indicated time points and analyzed by western blotting for pT662 and total Ypk1 levels.

A unique and distinguishing feature of ceramides in budding yeast is their C_26_-VLCFA. VLCFAs are produced through a four-step elongation cycle of shorter fatty acids, typically ranging from C_16_ to C_18_ in length. In budding yeast, the Elo1 elongase extends the C_12_–C_16_ fatty acyl-CoAs to the C_16_–C_18_ fatty acids (Oh et al., 1997). Elo2 is the rate-limiting enzyme in the elongation of fatty acids to VLCFA (Olson et al., 2015a) using shorter chain fatty acid substrates (C_16_–C_18_) and elongates them to acyl chains maximally containing 24 carbon atoms (C_24_). Elo3, in turn, uses saturated fatty acids of C_18_ as substrates to synthesize VLCFAs in the range of C_20_ to C_26_. Ceramide is formed from the direct condensation of VLCFA with an LCB by the ceramide synthases Lac1 and Lag1 (Fig. 1A).

Despite the established link between sphingolipid abundance and TORC2 activity, the precise lipid moiety, acyl-chain variations or hydroxylation status of ceramides that dictate TORC2 output and cell size regulation remains poorly understood. It remains unclear whether the signaling relies on sensing bulk lipid levels or recognizing specific structural motifs to couple cell size with nutrient availability. Recent advances have started to delineate the biophysical requirements necessary for this sensing process (Aguilera-Romero et al., 2014, 2023; Alcaide-Gavilán et al., 2018; Flor-Parra et al., 2022; Harayama and Riezman, 2018; Kellogg and Levin, 2022; Lucena et al., 2018; Rodriguez-Gallardo et al., 2020; Tettamanti et al., 2024 preprint). For example, loss of the major ceramide synthase activity in budding yeast hyperactivates TORC2 signaling despite adding LCB exogenously, which points to ceramide production as a key signaling molecule (Lucena et al., 2018).

In this study, we define the structural requirements of ceramides for the regulation of cell size and TORC2 signaling in *S. cerevisiae*. Central to this regulation is the ability of wild-type (WT) cells to actively reduce their volume upon transition to nutrient-poor conditions, a process typically driven by a concomitant reduction in TORC2 activity (Lucena et al., 2018). By systematically engineering ceramide acyl-chain composition, utilizing endogenous mutants and a heterologous panel of mammalian ceramide synthases, we investigated how the cell interprets different ceramide species during nutrient-dependent size modulation. Our results suggest that while long (C_24_–C_26_) hydroxylated ceramides are the physiological norm for robust TORC2 signaling, shorter C_18_–C_22_ chains can also permit normal size remodeling and responsive TORC2 dephosphorylation (pT662). However, this responsiveness is highly dependent on the genetic context and the specific synthase used. Strikingly, we found that ceramides produced by GhLag1 (C_18_), CerS2 (C_22_–C_24_), and in the *elo3*Δ (C_24_) background led to persistent pT662 signaling, effectively decoupling the regulation of cell size from the control of TORC2's basal activity. These findings reveal an unexpected complexity in lipid signaling: the nutrient-responsive state of TORC2 is not determined by chain length alone, but by a specific ceramide configuration that is disrupted in these uncoupled mutants, suggesting that ceramide architecture acts as a filter for downstream cellular responses.

## RESULTS

### Elo3-dependent VLCFA elongation contributes to nutrient-dependent regulation of cell size and is associated with altered TORC2 signaling

Our previous work showed that alterations in ceramide synthesis leads to significant defects in cell size and growth rate (Flor-Parra et al., 2022; Lucena et al., 2018). This initiates a response to enhance ceramide production through a TORC2-dependent pathway that involves the conserved SGK kinases Ypk1 and Ypk2 and the phosphatase PP2A through its regulatory subunit Rts1 (Alcaide-Gavilán et al., 2018; Lucena et al., 2018).

Ceramide biosynthesis requires an amide bond between an LCB and a VLCFA. The synthesis of the VLCFAs required for ceramide production comprises a specialized elongation cycle involving Elo1, Elo2, and Elo3. While Elo2 is the rate-limiting enzyme responsible for elongating fatty acids up to C_24_, Elo3 is unique in its ability to catalyze the final extension to C_26_ (Oh et al., 1997).

Since *elo1*Δ exhibited an overall fatty acid composition similar to that of a WT and contains normal amounts of VLCFAs (Dittrich et al., 1998), we focused on Elo2 and Elo3 mutants. To investigate the role of VLCFA length in this homeostatic response, we focused on Elo2 and Elo3 mutants. In budding yeast, cells control the ability to reduce their physical volume when shifted from a rich carbon source (YPD) to a poor or non-fermentable carbon source (YPG/E). This size reduction is physiologically coordinated with a decrease in TORC2 signaling activity, effectively matching the biosynthetic demands to nutrient availability (Lucena et al., 2018). In a rich carbon source (YPD), *elo2*Δ cells showed a small but consistent reduction in cell size compared to WT, while *elo3*Δ cells were comparable in size to WT. When shifted to a poor carbon source (YPG/E), *elo2*Δ cells exhibited an attenuated size reduction compared to WT, failing to achieve the standard physiological volume for these conditions. However, Elo3 was found to be essential for the nutrient modulation of cell size, since *elo3*Δ cells failed to adjust their size (Fig. 1B). Interestingly, *elo3*Δ cells exhibited improved colony proliferation at 25°C on poor carbon sources compared to rich sources (Fig. 1C).

We next analyzed TORC2 activity using a phosphospecific antibody (pT662) that recognizes a canonical TORC2-dependent phosphorylation site on its substrates, Ypk1 and Ypk2. The pT662 site is a canonical TORC2 target and widely used as a readout (Niles et al., 2012). In WT cells, TORC2 signaling is robustly downregulated upon transition from rich (YPD) to poor (YPGE) carbon sources. We observed that VLCFA elongation is required for maintaining proper TORC2 activity levels in both conditions. In rich media (YPD), both *elo2*Δ and *elo3*Δ mutants exhibited basal hyperactivation of TORC2, with pT662 levels significantly higher than WT (1.66±0.26 s.e.m. and 1.80±0.09 s.e.m. relative to WT, respectively) (Fig. 1D). Furthermore, these mutants failed to properly downregulate signaling upon nutrient shift. In YPGE, *elo2*Δ cells maintained pT662 levels that are significantly higher than the WT baseline [1.82±0.04 s.e.m. relative to WT; *P*=0.0305, analysis of variance (ANOVA)].

To further characterize the uncoupling of TORC2 signaling in the absence of C_26_, we monitored the kinetics of pT662 dephosphorylation following an acute nutrient shift. In WT cells, TORC2 activity dropped rapidly within 10 min of transition to YPGE (Fig. 1E). In contrast, *elo3*Δ mutants maintain high levels of pT662 for the duration of the time course. This kinetic analysis confirms that VLCFA length is critical for the rapid adjustment of TORC2 signaling to environmental changes.

Overall, these findings indicate that alteration of VLCFAs is sufficient to trigger TORC2 activation, but only the accumulation of C_20_ to C_24_ in *elo3*Δ cells is crucial for the nutrient-dependent regulation of cell size.

### Expression of mammalian ceramide synthase homologs differently impacts cell size and TORC2 signaling

**Fig. 2.**
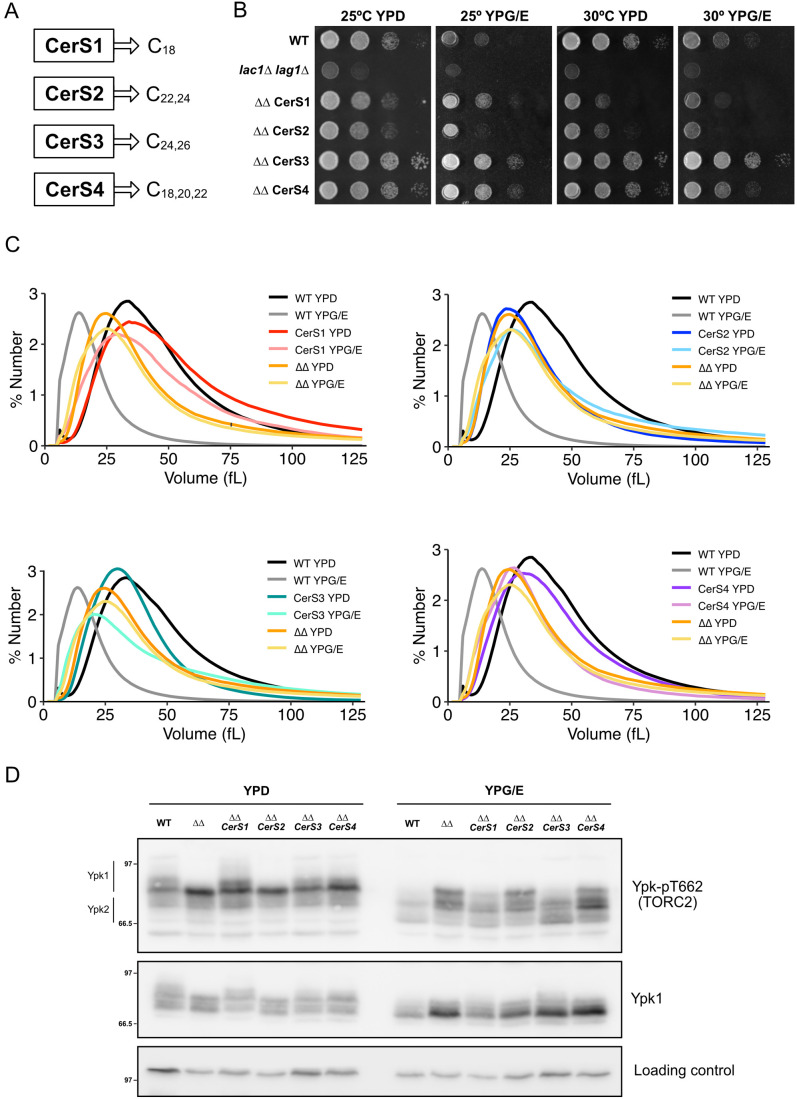
**Mammalian CerS isoforms reconstitute distinct ceramide species and produce differential growth and size phenotypes in yeast.** (A) Fatty acyl-CoA chain-length specificities of CerS1, CerS2, CerS3 and CerS4 according to Hannich et al. (2019). (B) A series of tenfold dilutions of the indicated strains were grown on YPD or YPG/E plates at 25°C and 30°C. All CerS isoforms rescue *lac1*Δ *lag1*Δ lethality; CerS1 and CerS2 expressions markedly reduce colony growth. (C) Cell size distributions of WT, *lac1*Δ *lag1*Δ and CerS-expressing strains in YPD and YPG/E. CerS2-expressing cells show a size reduction and loss of nutrient-dependent size modulation, phenocopying *elo3Δ*. CerS3 and CerS4 produce near-normal size profiles. Each plot is the average of three biological replicates. (D) Cells of the indicated genotypes were grown at 25°C to log phase in YPD or YPG/E medium. Ypk-pT662 and total Ypk1 were assayed by western blotting. A nonspecific background band was used as a loading control.

To further investigate the role of ceramide chain length, we decided to express mammalian ceramide synthases in yeast. In mammalian cells, six ceramide synthases (CerS1–CerS6) have been described so far (Guillas et al., 2003; Levy and Futerman, 2010; Venkataraman and Futerman, 2000). Unlike the yeast machinery, which produces a limited range of acyl chains, these mammalian isoforms display strict specificity toward fatty acyl-CoAs of defined lengths. Consequently, the expression of CerS homologs in budding yeast resulted in the production of ceramides and sphingolipids with different lengths of the fatty acid chain [Levy and Futerman (2010) and Fig. 2A]. We focused on ceramide synthases that synthesize major species of fatty acyl-CoA and ceramides ranging from C_18_ to C_26_.

The functional expression of the mammalian CerS isoforms was verified by their ability to rescue the growth defects of the *lac1*Δ *lag1*Δ double mutant lacking endogenous ceramide synthase. All four mammalian CerS rescued the *lag1*Δ *lac1*Δ lethality and produced distinct growth and size phenotypes (Fig. 2B and Hannich et al., 2019), indicating functional output despite species differences. However, strains expressing CerS1 (C_18_ specificity) and CerS2 (C_22_–C_24_) grew slower than those expressing CerS3 (C_24_–C_26_) and CerS4 (C_18_–C_22_). Notably, the CerS1- and CerS2-expressing strains showed a drastic reduction in colony growth in both rich and poor media (Fig. 2B).

Analysis of cell volume revealed striking differences. While CerS1 was similar to WT in rich media, CerS3 and CerS4 expression resulted in a cell size slightly smaller than that of WT cells. In contrast, expression of CerS2 caused a dramatic decrease in cell size, yielding cells as small as the *lag1*Δ *lac1*Δ mutant (Fig. 2C). Furthermore, CerS1, CerS3 and CerS4 reduced their size in response to poor nutrients, similar to *elo2*Δ. Surprisingly, the CerS2 mutant completely failed to modulate its size, similar to our observation with *elo3*Δ mutants (Figs 2C, 1B).

Next, we determined whether these cell size and growth phenotypes correlated with TORC2 activity. The expression of ceramide synthases from mammals was found to have a measurable impact on TORC2 activity to some extent. We found that expression of CerS2, much like the *lag1*Δ *lac1*Δ mutant, caused a significant increase in TORC2-dependent pT662 phosphorylation in both rich and poor media (Fig. 2D). We also observed a different phosphorylation state of Ypk1 in CerS2 cells in rich media and poor media, similar to the pattern observed in *lac1*Δ *lag1*Δ cells. This may be attributed to the activity of Fpk1/Fpk2 kinases, two redundant kinase paralogs whose roles in regulating Ypk1/2 remain not well understood (Roelants et al., 2010) (Fig. 2D).

**Fig. 3.**
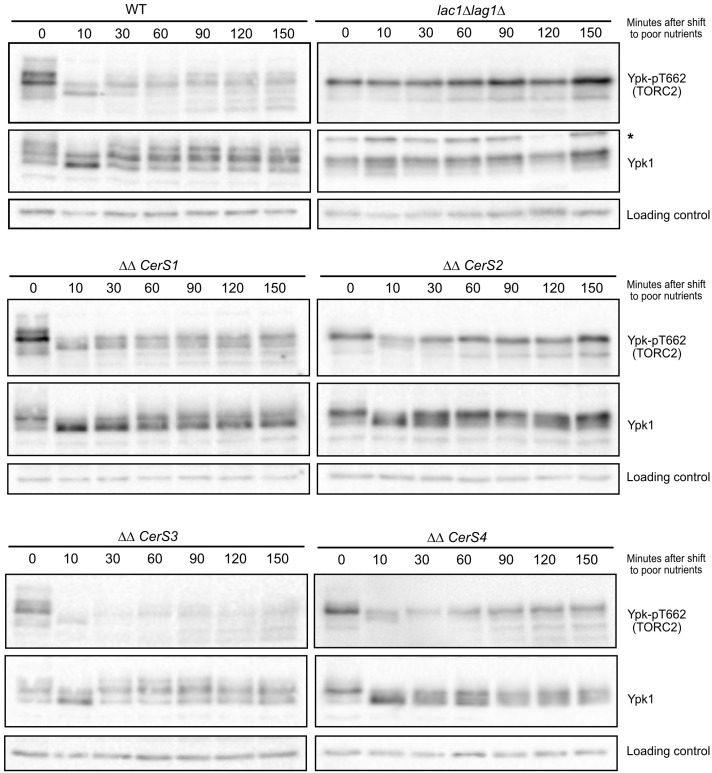
**Kinetic analysis of TORC2 signaling in yeast strains expressing mammalian CerS homologs.** Yeast strains expressing individual mammalian ceramide synthases (CerS1, CerS2, CerS3, and CerS4) in a *lac1*Δ *lag1*Δ background, along with WT and *lac1*Δ *lag1*Δ controls, were grown in rich media (YPD) and shifted to poor media (YPGE) at the indicated time points. Asterisk indicates non-specific background bands.

To investigate whether the length of ceramides produced by different mammalian ceramide synthases can restore nutrient-sensitive signaling in yeast, we performed acute nutrient shift experiments. In WT cells, TORC2-dependent phosphorylation of Ypk1 (pT662) drops rapidly within 10 min of shifting from rich (YPD) to poor (YPGE) carbon sources (Fig. 3). Consistent with our previous results (Lucena et al., 2018), *lac1*Δ *lag1*Δ null mutants showed high basal signaling that remained persistent throughout the 150-min time course, confirming that ceramide synthesis is essential for TORC2 downregulation.

Among the mammalian homologs, CerS1, CerS4 and, to some extent, CerS3 successfully restored the nutrient-dependent response, with pT662 levels dropping sharply following the shift, mirroring the WT kinetic profile. In contrast, cells expressing CerS2 exhibited a complete failure to downregulate TORC2 signaling. In the CerS2 background, pT662 levels remained constitutively high and unresponsive to the nutrient shift. These results demonstrate that the ability of TORC2 to sense and respond to nutrient stress is not a generic property of ceramide synthesis but is specifically dependent on the production of a physiological acyl-chain profile.

Taken together, these nutrient shift profiles reveal a striking functional parallel between the mammalian CerS2-expressing cells and the yeast elongase mutants *elo3*Δ.

### GhLag1 ceramide decouples cell size modulation from TORC2 signaling

**Fig. 4.**
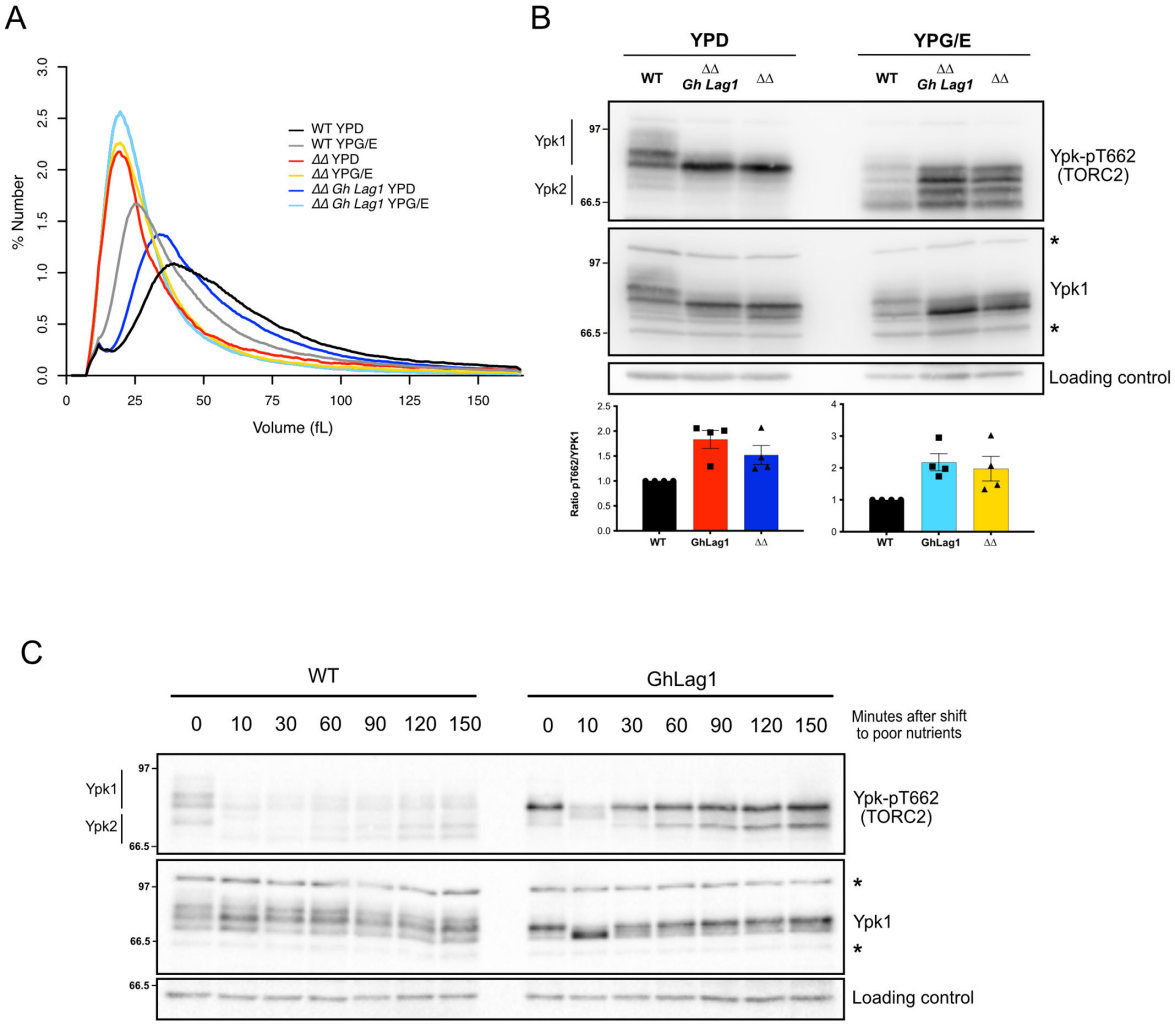
**C_18_-ceramide-producing GhLag1 cells decouple TORC2 signaling from nutrient-dependent cell size control.** (A) Cells of the indicated genotypes were grown to log phase at 25°C and cell-size distributions were determined using a Coulter counter. Each plot is the average of three biological replicates. (B) WT, *lac1*Δ *lag1*Δ and GhLag1 cells were grown to early log phase in YPD or YPG/E at 25°C. Ypk-pT662 and total Ypk1 were assayed by western blotting. An asterisk indicates a non-specific band on the Ypk1 antibody. Relative phosphorylation levels of Ypk1 at the TORC2-dependent site (T662) were determined by western blotting analysis. Signals were normalized to total protein loading and expressed relative to the WT control within each carbon source. Data represent the mean±s.e.m. of four independent biological replicates. Statistical significance was determined by one-way ANOVA. (C) Time course of Ypk1/2-pT662 phosphorylation in WT and GhLag1 cells after a shift from YPD to YPG/E. Asterisks indicate non-specific background bands used as loading controls.

We also explored the evolutionary conservation of this pathway by expressing a codon-optimized cotton Lag1 homolog (GhLag1) in a yeast strain lacking endogenous ceramide synthases. This previously characterized strain produces almost exclusively C_18_ ceramides (Epstein et al., 2012). Similar to other shorter-ceramide mutants, GhLag1 cells showed a consistent decrease in cell size in rich media (Fig. 4A). Interestingly, and in contrast to *elo3*Δ and CerS2 mutants, GhLag1 cells were fully capable of adjusting their size in response to a poor carbon source (Fig. 4A).

This result was particularly surprising because, despite its normal size modulation, the GhLag1 mutant exhibited highly elevated basal TORC2 signaling (pT662), comparable to the *lag1*Δ *lac1*Δ null mutant, in both rich and poor media (Fig. 4B). Quantification of pT662 levels revealed that while WT cells downregulate TORC2 signaling upon shifting to poor nutrients, cells expressing GhLag1 maintain significantly higher activity (2.18±0.27 s.e.m. relative to WT; *P*=0.0312, ANOVA). Notably, this hyperactivation was also present in rich media (1.84±0.18 s.e.m. relative to WT; *P*=0.0094), indicating that C_18_ ceramide production results in a constitutive uncoupling of the TORC2-nutrient-sensing axis (Fig. 4B).

We next tested whether TORC2 is modulated in response to rapid changes in the carbon source. In WT cells, a shift to a poor carbon source caused rapid loss of TORC2-dependent Ypk1/2 phosphorylation. As cells adapted to the new carbon source, TORC2 activity recovered but remained below levels observed in rich carbon (Lucena et al., 2018). Thus, we analyzed the acute signaling response to a nutrient shift in GhLag1 cells. In WT cells, shifting from rich to poor media causes a rapid and substantial decrease in TORC2 signaling. While GhLag1 cells showed a normal initial drop in pT662, they failed to sustain this response, and TORC2 signaling rapidly recovered to abnormally high levels (Fig. 4C).

Together, these results indicate that C_18_ ceramides are sufficient to trigger the nutrient-dependent cell size modulation response. However, they are insufficient to properly regulate the basal activity or the acute nutrient-responsive dynamics of the TORC2 signaling network.

### Differential roles of sphingoid-base and fatty acid hydroxylation in cell size control

To determine if the position of the hydroxyl modification within the ceramide structure dictates its signaling function, we investigated the roles of Sur2 and Scs7 hydroxylases. We compared cell growth, cell size, and TORC2 signaling profiles of single- and double-deletion mutants in rich (YPD) and poor (YPG/E) carbon sources.

**Fig. 5.**
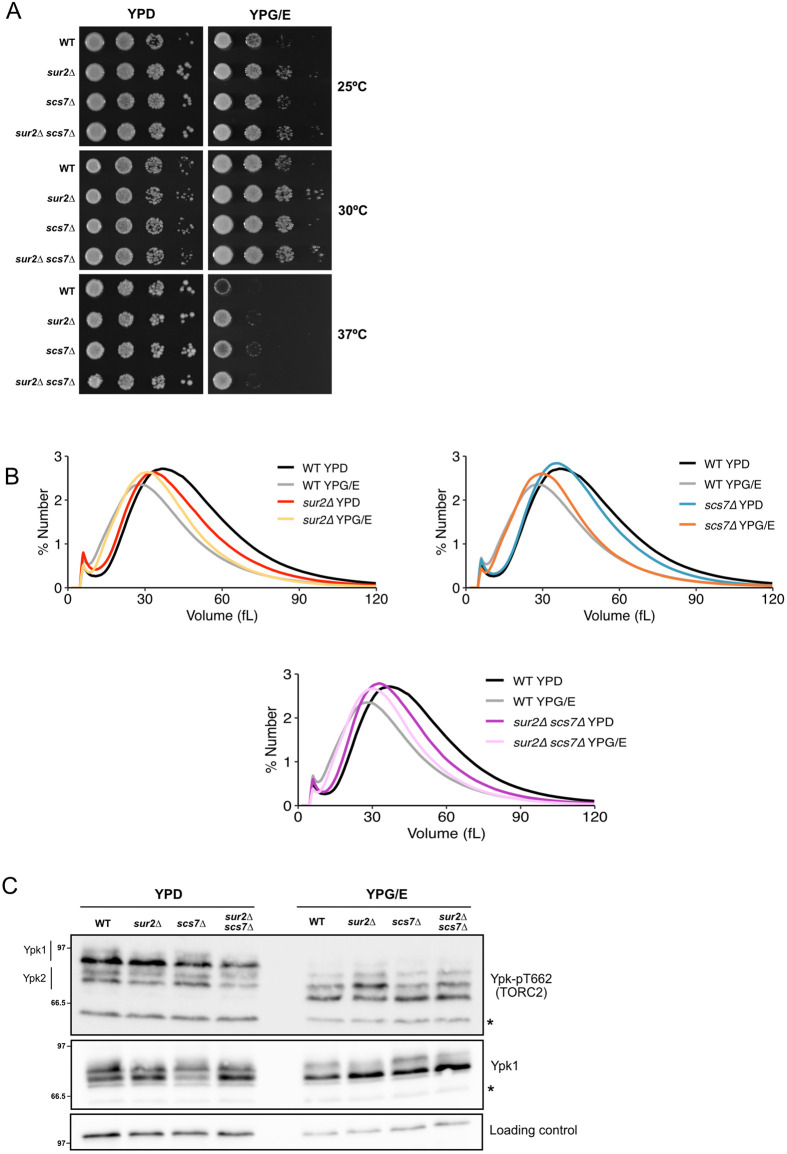
**Differential requirements of sphingoid-base and fatty acid hydroxylation for cell growth, size homeostasis, and TORC2 signaling.** (A) Analysis of cell growth. WT, *sur2*Δ, *scs7*Δ, and double-mutant *sur2*Δ *scs7*Δ strains were grown to exponential phase, and tenfold serial dilutions were spotted onto rich (YPD) or poor (YPG/E) media plates. Plates were incubated at the indicated temperatures (25°C, 30°C and 37°C) for 2–3 days. (B) Coulter counter cell size analysis. The indicated strains were grown to log phase in YPD or YPG/E. Cell volume distributions (fL) were measured using a Coulter counter as described in the Materials and Methods. Each plot is the average of three biological replicates. (C) Assessment of TORC2 signaling activity. Whole-cell extracts from strains grown in YPD or YPG/E were analyzed by western blotting using phosphospecific Ypk1-pT662 and total anti-Ypk1 antibodies. Asterisks indicate nonspecific background bands used as loading controls.

We first assessed cellular growth via serial dilution assays (Fig. 5A). The *sur2*Δ and *scs7*Δ mutants displayed better growth to non-fermentable carbon sources, a phenotype reminiscent of the *elo2*Δ mutant. All mutants also showed a slightly better response to high temperature (37°C). The double mutant *sur2*Δ *scs7*Δ phenocopied the response of the *sur2*Δ single mutant, indicating that the lack of base hydroxylation is the dominant stress determinant.

Next, we examined cell size regulation using Coulter counter analysis (Fig. 5B). The *scs7*Δ mutant displayed a cell size profile comparable to WT. Importantly, these cells retained the ability to modulate their volume, maintaining the ability to perform nutrient modulation of cell size when transferred from YPD to YPG/E. This demonstrates that hydroxylation of the fatty acid is dispensable for nutrient-dependent size remodeling. In contrast, *sur2*Δ cells exhibited a small cell size in rich media and failed to undergo further size reduction in poor media, similar to what we observed in *elo2*Δ cells. Again, the *sur2*Δ *scs7*Δ double mutant showed a size distribution identical to the *sur2*Δ single mutant, confirming that the loss of Sur2 partially prevents size modulation regardless of the Scs7 status.

Finally, we analyzed TORC2 pathway activity (Fig. 5C). Quantification of the western blotting revealed that the loss of neither Sur2 nor Scs7 resulted in statistically significant alterations to TORC2 signaling compared to WT. Despite the severe failure of *sur2*Δ mutant to modulate their cell size, TORC2 signaling remained nutrient-responsive, dropping appropriately upon shift to YPG/E.

These results establish a clear dissociation between TORC2 signaling and the physical execution of cell size control. Because the signaling machinery detects and responds to the nutrient shift normally in *sur2*Δ mutants, their failure to reduce volume must be attributed to a biophysical defect in the membrane itself rather than a failure in sensory perception. We conclude that while TORC2 activity is sensitive to acyl-chain length, sphingoid-base hydroxylation is a downstream requirement essential for the structural execution of cell size remodeling.

## DISCUSSION

The intricate diversity of lipid structures is fundamental to their function, yet how specific structural variations impact complex cellular processes like growth control remains a key question (Futerman and Hannun, 2004; Harayama and Antonny, 2023; Tani, 2024). In this study, we provide evidence that the structural integrity of ceramides, specifically their acyl-chain length and hydroxylation, is a critical input for the nutrient-sensing TORC2 pathway and the regulation of cell size in *S. cerevisiae*. By systematically manipulating acyl-chain length, hydroxylation state, and biosynthetic origin of ceramides, we identify the specific lipid features required for nutrient modulation of cell growth and for the proper tuning of TORC2 activity. Our findings support a model where the cell monitors its metabolic state by sensing the presence of mature, correctly structured ceramides and sphingolipids, which in turn dictates decisions on cell growth and proliferation.

Our genetic analysis of VLCFA elongases demonstrates that the final Elo3-dependent elongation step, producing C_26_ acyl chains, is essential for cell size reduction under poor nutrient conditions. Although both *elo2*Δ and *elo3*Δ mutants displayed elevated TORC2 signaling, only *elo3*Δ cells failed to adjust their size upon nutrient limitation. This uncoupling indicates that TORC2 hyperactivation alone cannot compensate for the absence of long-chain ceramides and suggests that specific C_24_–C_26_ acyl chains are biophysically required for membrane remodeling. This result suggests that membranes lacking C_26_ ceramides are perceived by the cell as being under stress, similar to the response observed during the lack of ceramide production (Lucena et al., 2018), from membrane tension changes (Berchtold et al., 2012; Riggi et al., 2018) and also from VLCFA synthesis (Olson et al., 2015a,b). The kinetic failure of *elo3*Δ to downregulate TORC2 signaling during an acute nutrient shift (Fig. 1E) further suggests that C_26_-VLCFAs are a requisite signal for the signaling machinery to enter a nutrient-limited state.

The expression of mammalian ceramide synthases reinforced this conclusion (Hannich et al., 2019). The expression of CerS3 (C_24_–C_26_) largely restored cell size and nutrient responsiveness to *lag1*Δ *lac1*Δ cells, although these cells remained slightly smaller than WT in YPD. In contrast, CerS2 (C_22_–C_24_) yielded a reduced cell size compared to WT and is incapable of nutrient-dependent size reduction. Notably, our nutrient shift profiles reveal a striking functional parallel between CerS2-expressing cells and the yeast *elo3*Δ mutant. Much like *elo3*Δ, CerS2 exhibited a total kinetic failure to downregulate pT662 signaling upon nutrient shift, remaining constitutively hyperactive (Fig. 3). This demonstrates that CerS2 expression does not merely fail to rescue *lac1*Δ *lag1*Δ mutant; it actively phenocopies the elongase-deficient state, reinforcing the conclusion that TORC2-nutrient sensing is strictly tuned to a physiological acyl-chain profile.

These observations reveal a structural threshold for function: ceramides shorter than C_24_ are interpreted as a stress signal, whereas C_24_–C_26_ acyl chains are required to maintain proper physiological responses. The structural basis for this C_26_ requirement is best explained by the molecular caliper mechanism identified for the Elo elongase family (Denic and Weissman, 2007). In this model, the chain length is determined by the physical distance between the elongase active site and a specific lysine residue on the luminal face of the ER, which acts as a stop signal for elongation. Our findings suggest that the TORC2–Ypk1 signaling axis is specifically tuned to the successful completion of this caliper-measured product. When this physical threshold is not reached, either through the deletion of *ELO3* or the expression of the shorter-chain-preferring CerS2, the resulting incomplete acyl chains (C_18_–C_24_) are sensed as a dominant membrane stress, leading to the kinetic failure of TORC2 downregulation we observe during nutrient shifts. Because all CerS constructs are expressed from the same TDH3 promoter, differences in phenotypes are unlikely to arise from major differences in expression level. However, we cannot exclude the possibility that variation in protein stability or activity contributes to the observed effects.

A striking finding of our study came from the GhLag1 mutant, which exclusively produces C_18_ ceramides (Epstein et al., 2012). These cells decoupled the two key phenotypes we measured: nutrient modulation of cell size and TORC2 signaling. While exhibiting highly elevated basal and persistent TORC2 signaling (2.18-fold higher than WT in YPGE), GhLag1 cells were, surprisingly, still able to reduce their size when shifted to a poor carbon source (Fig. 4). This uncoupling suggests that C_18_ ceramides can support the physical execution of size remodeling even when the signaling machinery remains in a hyperactive state.

The contradictory behavior of the two C_18_-producing enzymes, GhLag1 and CerS1, revealed an unexpected layer of complexity. While CerS1-expressing cells maintain a WT-like kinetic drop in TORC2 signaling upon shift, GhLag1 signaling remains constitutively high and recovers rapidly after an initial transient drop (Fig. 4C). We propose that this divergence arises from distinct substrate specificities for the LCB or different levels of subsequent hydroxylation (Mullen et al., 2012). This model suggests that shorter ceramides can support nutrient modulation of cell size only when they possess the critical C4-hydroxyl group. Notably, CerS1 is structurally and functionally distinctive from the other CerS and is found on an entirely separate branch of the phylogenetic tree (Pewzner-Jung et al., 2006).

Our analysis of hydroxylation mutants allowed us to distinguish the specific lipid features driving nutrient modulation of cell size from those responsible for adjusting TORC2 signaling output. Notably, we found that neither Scs7-dependent fatty acid hydroxylation nor Sur2-dependent base hydroxylation significantly impacts the amplitude or responsiveness of TORC2 signaling. Although *sur2*Δ and *scs7*Δ backgrounds alter the lipid profile, they do not interfere with the primary sensing of nutrient status by the TORC2–Ypk1 axis.

In contrast, the loss of Sur2-mediated sphingoid-base hydroxylation revealed a fundamental dissociation between signaling and phenotype. *sur2*Δ mutants show decreased cell size in rich media as previously described (Deng et al., 2025; Pewzner-Jung et al., 2006) and phenocopy the size-modulation defects of *elo3*Δ, yet they retain a normal TORC2 signaling response to nutrient shift. We conclude that the defect observed in *sur2*Δ cells may represent a failure of biophysical competence rather than sensory perception; the signaling machinery detects the nutrient shift, but the membrane architecture is unable to execute the required morphological change.

Altogether, our findings support a model in which yeast cells monitor the structural integrity of ceramides, rather than their bulk levels, to coordinate membrane composition with growth decisions. Mature C_26_-PHCers, defined by a long acyl chain and a hydroxylated LCB, act as a molecular signal of membrane health. Deviations from this molecular signature, such as the accumulation of C_18_–C_24_ species in *elo3*Δ or CerS2 backgrounds, keep TORC2 in a constitutively active state and impair cell size control.

This structural model explains how TORC2 converts subtle lipid structural features into distinct physiological responses and raises new questions about the molecular sensors responsible for detecting ceramide structure. Our work establishes that ceramide structure is a critical determinant of TORC2 signaling and nutrient modulation of cell size control in budding yeast. By identifying the specific structural motifs required for proper function, we reveal how membrane lipid architecture is translated into actionable growth signals. Future studies aimed at identifying the molecular machinery that senses ceramide structure and linking these lipids to TORC2 regulation will provide fundamental insight into conserved principles of lipid-based signaling across eukaryotes.

## MATERIALS AND METHODS

### Yeast strains and culture conditions

All strains are in the *S. cerevisiae* W303 background (leu2-3,112 ura3-1 can1-100 ade2-1 his3-11,15 trp1-1 GAL+ssd1-d2). Table 1 shows additional genetic features and backgrounds. One-step PCR-based gene replacement was used for making deletions at the endogenous locus (Janke et al., 2004; Longtine et al., 1998). Cells were grown in YP medium (1% yeast extract, 2% peptone, 40 mg/l adenine) supplemented with 2% dextrose (YPD) or 2% glycerol/ethanol (YPG/E). To analyze cells shifted from rich to poor nutrients (Figs 1E, 3, 4C), cultures were grown in YPD medium overnight at 25°C to an OD_600_ of less than 0.8. After adjusting optical densities to normalize protein loading, cells were washed three times with a large volume of YPG/E medium and then incubated at 30°C in YPG/E for the time course. At each time point, 1.6 ml of samples was collected.

**Table 1.** Strains used in this study

Strain	MAT	Genotype	Source
RL1	a	*bar1*	Douglas Kellogg
RL327	a	*elo2*Δ*::*KanMX	This study
RL337	a	*elo3*Δ*::*KanMX	This study
AL378	α	*lag1*Δ::HIS3 *lac1*Δ::ADE2	Howard Riezman (RH7165)
AL379	α	RH7165 TDH3pr-CERS1-CYC1term TRP1	Howard Riezman (RH7911)
AL380	α	RH7165 TDH3pr-CERS2-CYC1term TRP1	Howard Riezman (RH7912)
AL381	α	RH7165 TDH3pr-CERS3-CYC1term TRP1	Howard Riezman (RH7913)
AL382	α	RH7165 TDH3pr-CERS4-CYC1term TRP1	Howard Riezman (RH7914)
RL190	a	*lag1*Δ::HIS3 *lac1*Δ::ADE2 TDH3::GhLAG1::TRP1	MMY1678
AL402	a	*sur2*Δ::HIS	This study
AL445	a	*scs7*Δ::HIS	This study
AL435	a	*sur2*Δ::HIS *scs7*Δ::KanMX	This study

Howard Riezman background: his3-11,15, leu2, trp1, ura3, ade2, bar1.

MMY1678 background: his4, leu2, ura3.

### Analysis of cell size and cell proliferation assays

Cell cultures were grown overnight to early log phase at 25°C. A 900 μl sample of each culture was fixed with 100 μl of 37% formaldehyde for 30 min and then washed twice with PBS containing 0.04% sodium azide and 0.02% Tween-20. Cell size was measured using a Coulter Counter Z3 (Channelizer Z3, Beckman Coulter) as previously described (Lucena et al., 2018). In brief, cells were diluted into a 10 ml diluent (Isoton II; Beckman Coulter) and sonicated for 3 s before cell sizing. Each plot is the average of three independent biological replicates, in which three independent technical replicates were averaged. Summary of mean, median and mode cell volumes (fL) measured by Coulter counter are summarized in supplemental Table S1. To assay the rate of cell proliferation on plates, cells were grown overnight in the indicated medium at 25°C and adjusted to an OD_600_ of 0.5. Tenfold serial dilutions were spotted onto YPD or YPG/E and incubated at 25°C, 30°C or 37°C for 3 days.

### Western blotting

For western blotting, cells growing in early log phase were grown overnight at 25°C to an OD_600_ of 0.6 as described by Lucena et al. (2024). After adjusting optical densities to normalize protein loading, 1.6 ml of each sample was collected and centrifuged at 20,000 ***g*** for 30 s. The supernatant was removed, and 250 μl of glass beads was added before freezing in liquid nitrogen. We collected 1.6 ml of samples at each time point. Cells were lysed into 140 μl of sample buffer (65 mM Tris HCl, pH 6.8, 3% SDS, 10% glycerol, 50 mM sodium fluoride, 100 mM β-glycerophosphate, 5% 2-mercaptoethanol, and bromophenol blue). Phenylmethylsulfonyl fluoride (PMSF) was added to the sample buffer to 2 mM immediately before use. Cells were lysed in a Mini-Beadbeater-16 (BioSpec) at top speed for 2 min. The samples were removed and centrifuged for 15 s at 20,000 ***g*** in a microfuge and placed in boiling water for 5 min. After boiling, the samples were centrifuged for 5 min at 20,000 ***g*** and loaded onto an SDS polyacrylamide gel. Samples were analyzed by western blotting as previously described (Harvey et al., 2011). SDS-PAGE gels were run at a constant current of 20 mA, and electrophoresis was performed on gels containing 10% polyacrylamide and 0.13% bis-acrylamide. Proteins were transferred to nitrocellulose using a Trans-Blot Turbo system (Bio-Rad). Blots were probed with a primary antibody overnight at 4°C. A rabbit anti-phospho-T662 antibody (a kind gift from Ted Powers, University of California, Davis) was used to detect TORC2-dependent phosphorylation of Ypk1/2 at a dilution of 1:20,000 in TBST (10 mM Tris-Cl, pH 7.5, 100 mM NaCl, and 0.1% Tween-20) containing 3% milk. Total Ypk1 was detected using an anti-Ypk1 antibody (a kind gift from Douglas Kellogg, University of California, Santa Cruz, USA; Alcaide-Gavilán et al., 2020) at a dilution of 1:10,000. All blots were probed with an anti-rabbit IgG Peroxidase AffiniPure goat secondary antibody (Jackson ImmunoResearch, 111-035-003) for 45–90 min at room temperature. Equal protein loading was confirmed by using a nonspecific background band detected by anti-Ypk1/anti-phospho-T662 antibodies as a loading control, as previously described (Lucena et al., 2018). This band serves as a reliable internal control, as its intensity remains constant across the experimental conditions and nutrient shifts tested.

### Statistical analysis

A minimum of two independent biological replicates was performed for each experimental condition. For the statistical analyses, a one-way ANOVA test was performed using Prism 10 (GraphPad). Error bars represent mean±s.e.m.

## Supplementary Material

10.1242/biolopen.062633_sup1 Supplementary information

Table S1.
