# Supplementary material for: Uncoupling of nutrient sensing and cell size control by specific defects in ceramide structure
Source: Biol Open. 2026 May 26;15(5):bio062633. doi: 10.1242/bio.062633 (PMC13267774; doi:10.1242/bio.062633)
Supplement: Supplementary information [file biolopen-15-062633-s1.pdf]

Table S1.

Available for download at
https://journals.biologists.com/bio/article-lookup/doi/10.1242/bio.062633#supplementary-data

Biology Open • Supplementary information
